# Innovative Approaches to Enhancing the Biomedical Properties of Liposomes

**DOI:** 10.3390/pharmaceutics16121525

**Published:** 2024-11-27

**Authors:** Ioana Lavinia Dejeu, Laura Grațiela Vicaș, Eleonora Marian, Mariana Ganea, Olimpia Daniela Frenț, Paula Bianca Maghiar, Flaviu Ionut Bodea, George Emanuiel Dejeu

**Affiliations:** 1 Department of Pharmacy, Faculty of Medicine and Pharmacy, University of Oradea, 29 Nicolae Jiga Street, 410028 Oradea, Romania; ioana.dejeu@gmail.com (I.L.D.); marian_eleonora@yahoo.com (E.M.); madafarm2005@yahoo.com (M.G.); daniela.olimpia@yahoo.com (O.D.F.); 2 Doctoral School of Biomedical Science, University of Oradea, 1 University Street, 410087 Oradea, Romania; pao.badea@gmail.com (P.B.M.); dr.flaviu.bodea@gmail.com (F.I.B.); 3 Department of Surgical Disciplines, Faculty of Medicine and Pharmacy, University of Oradea, 10 Piata 1 Decembrie Street, 410073 Oradea, Romania; dejeu.george@gmail.com

**Keywords:** liposomes, cancer, bioavailability, nanoparticles, nanomedicine

## Abstract

Liposomes represent a promising class of drug delivery systems that enhance the therapeutic efficacy and safety of various pharmaceutical agents. Also, they offer numerous advantages compared to traditional drug delivery methods, including targeted delivery to specific sites, controlled release, and fewer side effects. This review meticulously examines the methodologies employed in the preparation and characterization of liposomal formulations. With the rising incidence of adverse drug reactions, there is a pressing need for innovative delivery strategies that prioritize selectivity, specificity, and safety. Nanomedicine promises to revolutionize diagnostics and treatments, addressing current limitations and improving disease management, including cancer, which remains a major global health challenge. This paper aims to conduct a comprehensive study on the interest of biomedical research regarding nanotechnology and its implications for further applications.

## 1. Introduction

Nanotechnology focuses on the application of therapeutic agents at the nanoscale, analyzing molecular interactions and their effects on macroscopic material properties. This discipline has significantly impacted treatments, particularly cancer, by developing liposome-based drug delivery systems [1].

Nanotechnology develops particles of varying dimensions from 1 to 100 nm, and by reducing particle size, long-term benefits, deeper skin penetration, and superior product quality emerge, which larger particles cannot match [2]. Additionally, nanotechnology enables the controlled release of substances from nanoparticle carriers based on interactions between components, substance content, and polymers, improving stability and allowing specific site targeting [3].

Nanotechnology offers the potential to utilize various nanoparticles (NPs) in the development of anticancer therapies, enabling a more efficient delivery of therapeutic and chemotherapeutic agents. Ongoing investigations and innovations in this domain are promising for enhancing patient outcomes. The application of emerging nanotechnologies in medicine can significantly advance clinical practice by addressing current limitations in the diagnosis, treatment, and management of the illness.

The latest approach involves incorporating the bioactive component into inert lipid carriers (oils, surfactants, and dispersions), self-emulsifying compositions, microemulsions, liposomes, niosomes, exosomes, ethosomes, and solid lipid nanoparticles, consequently enhancing the solubility and bioavailability of lipophilic drugs [4]. Nanoparticles can be categorized into three primary categories: organic (polymers and lipid nanoparticles), inorganic (gold nanoparticles, carbon nanotubes, iron oxide nanoparticles, and silica nanoparticles), and biological (exosomes), each having certain advantages and disadvantages [5,6,7,8,9,10,11,12].

Liposomes are small, artificially engineered round vesicles with one or more bilayers, capable of encapsulating a diverse array of lipophilic and hydrophilic substances [4,13].

They have appeared as a highly successful drug delivery technology in the biomedical field, owing to their capacity to encapsulate hydrophobic and hydrophilic substances [1]. Given their versatile physicochemical and biophysical properties, liposomes are being extensively studied as a critical delivery mechanism for active substances, showing significant promise. Liposome approaches effectively transport therapeutic agents to disease sites by utilizing the enhanced permeability and retention effect, resulting in improved treatment efficacy [14]. This specific delivery enables the precise direction of molecules to affected cells or tissues, minimizing adverse reactions and enhancing therapeutic efficacy [15].

Cancer remains an exceptionally dangerous disease, responsible for 1.8 million deaths worldwide. Although significant advances have been made in traditional treatments, these approaches have not yet achieved complete eradication of the disease [16].

Liposomes are being intensively studied due to their effectiveness in cancer treatment and are regarded as promising drug delivery systems (DDSs) owing to their distinctive characteristics, including the high entrapment efficiency of active substances, accessibility, and scalability in production. However, their application is limited by the active substance’s rapid release and the ability to modify its surface.

Lipid vesicles exhibit strong biocompatibility and biodegradability, making them highly effective in biomedical applications [17].

### The History and Liposome’s Generations

The history of liposomes showcases their evolution from a basic scientific discovery to a powerful tool in medicine. With ongoing research and innovation, liposomal technology continues to expand, offering new possibilities for drug delivery and therapeutic applications.

The term “liposome” originates from two Greek words: “lipos”, meaning fat, and “soma”, which means body. This term is connected to the structural components of the body, specifically phospholipid molecules [18]. A liposome can be described as a spherical vesicle that features a membrane made up of phospholipid bilayers, similar to the membranes of cells [19]. The concept of liposomes was first introduced in 1961 by British hematologist Dr. Alec D. Bangham at the University of Cambridge [20].

In the early 1970s, researchers began to characterize liposomes more extensively, exploring their properties and behaviors. G. Gregoriadis was the first to propose the use of liposomes as carriers for drug delivery to cell membranes [21].

In the 1980s, researchers focused on improving the stability and efficacy of liposomal formulations. In 1986, Doxil, a liposomal formulation of doxorubicin, was approved by the FDA for the treatment of cancer. This marked a significant milestone, demonstrating the effectiveness of liposomes in delivering chemotherapeutic agents while reducing toxicity.

In the 1990s, the introduction of PEGylation (the attachment of polyethylene glycol (PEG) chains to liposomes) significantly improved their pharmacokinetics and biocompatibility.

In the 2000s Era, also known for the expansion of applications, liposomal formulations expanded beyond oncology to include vaccine delivery, gene therapy, and the treatment of infectious diseases. For example, liposomes were utilized in the development of vaccines for diseases such as hepatitis B.

Research in 2010 focused on optimizing liposomal formulations for specific applications, such as targeted delivery, improved pharmacokinetics, and reduced side effects. Advances in nanotechnology allowed for the design of liposomes with specific targeting capabilities, such as ligand-conjugated liposomes.

In 2020, the COVID-19 pandemic accelerated research into liposomal formulations for mRNA vaccines, such as the Pfizer-BioNTech and Moderna vaccines, which utilize lipid nanoparticles (similar to liposomes) to deliver mRNA.

Liposomes have been synthesized using various methods and modified to optimize their properties, including their surface charge, size, number of layers, and circulation in biological fluids [22,23]. Conventional drug delivery methods have suffered from drawbacks including inadequate targeting and diminished therapeutic indices, resulting in adverse effects, increased costs, and prolonged therapy duration. To address these challenges, nano-delivery devices have been created for various medicinal purposes; thus, liposomes have been extensively explored for therapeutic applications to enhance therapeutic efficacy and safety for various active substances [24,25,26]. Colloidal delivery systems offer an effective method to protect and transport polyphenols, contributing to the prevention of degradation, enhancing stability in biological contexts, and facilitating targeted absorption [27]. Moreover, the combination of various polyphenols or their conjunction with other bioactive chemicals is seen as a viable approach to mitigate the issues of limited bioavailability [28,29].

Second-generation liposomes named “stealth liposomes” or “PEGylated liposomes” are characterized by the incorporation of polyethylene glycol or other hydrophilic polymers onto the surface of the liposomes [30]. This helps to prolong their circulation time in the body, enhancing their ability to reach target sites, improving their overall pharmacokinetic profile, and reducing their recognition and clearance by the immune system [31]. These modifications result in superior pharmacokinetics, enhanced biodistribution, decreased toxicity, and heightened therapeutic efficacy [32]. This PEGylation process helps to prevent rapid clearance by the body’s reticuloendothelial system (RES) [33], which would otherwise reduce the effectiveness of the treatment by shortening the time these nanomaterials remain in the bloodstream [34]. PEGylation can also enhance the stability of liposomes against aggregation and fusion, which is particularly important in physiological conditions. They can facilitate the delivery of drugs to target tissues more effectively [35]. The most frequently used polymers in liposomal formulations are polyethylene glycol, chitosan, and polydopamine [36].

The third generation of liposomes is commonly known as “smart”, “stimuli-responsive”, or “targeted” liposomes. These liposomes are designed to respond to specific environmental triggers, such as changes in pH, temperature, or the presence of certain biomolecules, which can trigger the release of their cargo or the activation of their functionalities at the target site [37].

Some examples of third-generation liposomal systems include the following:

- pH-sensitive liposomes that release their payload in response to the acidic environment of the tumor microenvironment or within endosomes [38];
- Thermosensitive liposomes are engineered to undergo a phase transition and release their contents upon exposure to mild hyperthermia, which can be induced using various heating modalities, such as focused ultrasound or radiofrequency ablation [39];
- Enzyme-triggered liposomes are designed to release their cargo when exposed to specific enzymes overexpressed in the target tissue, such as matrix metalloproteinases in the tumor microenvironment [40].

Furthermore, the third generation of liposomes may also include the development of hybrid lipid-polymer nanoparticles, where the liposomal membrane is combined with a polymeric core to create a more stable and versatile drug delivery system.

These advanced liposomal systems represent the forefront of liposome research and development, offering unprecedented levels of control and precision in targeted drug delivery and other biomedical applications [38,41,42].

## 2. Structural Components of Liposomes

Conventional liposomes, with dimensions ranging from 50 to 1000 nm, are used locally in dermatology, oral, systemic, and inhalation drug delivery, reducing toxicity and enhancing the drug’s effectiveness. They are composed of both structural and non-structural components. Phospholipids and cholesterol represent the major structural components of liposomes [43,44,45].

### 2.1. Phospholipids

Biological membranes are primarily composed of phospholipids, which are classified into two types: phosphoglycerides and sphingolipids. Natural phospholipids include phosphatidylcholine, phosphatidylethanolamine, and phosphatidylserine, while synthetic ones include dioleoyl phosphatidylethanolamine, dioleoyl phosphatidylcholine, and distearoyl phosphatidylcholine [46,47]. The predominant phospholipids are phosphatidylcholine and phosphatidylethanolamine. Since phosphatidylcholine molecules are insoluble in water, they compact tightly together to reduce adverse interactions among their long hydrophobic hydrocarbon chains and the surrounding aqueous environment, resulting in planar bilayers. Glycerophospholipids, which can make up more than 50% of the membrane lipids, are commonly found in liposomal formulations.

Phospholipids have a structure consisting of a hydrophilic component made up of choline, a phosphate group, and glycerol and a hydrophobic component with two branches formed from essential fatty acids [48].

The choice of lipid bilayer components influences the charge and permeability of the vesicles. Less stable accessible bilayers of the vesicles can be synthesized utilizing unsaturated phosphatidylcholine variants derived from natural sources. The rigid, impermeable bilayer structure is achieved by the utilization of saturated phospholipids containing long acyl chains. Closed structures are formed by the hydration of phospholipids in aqueous solutions [49]. Depending on the aqueous or lipid nature of the drug, they are translocated across the cell membrane via one or more phospholipid bilayers. A series of phospholipids are used for the production of liposomes: phosphatidylcholine, phosphatidylethanolamine, phosphatidylserine, phosphatidylinositol, and phosphatidylglycerol.

### 2.2. Cholesterol

Cholesterol can be present in membranes in large amounts without forming a bilayer structure in a molar ratio of 1:1 or 2:1 relative to phosphatidylcholine [47,50].

It is situated in the center of the membrane, aligned with the acyl chains, with the hydroxyl group oriented toward the aqueous area. The ability to dissolve cholesterol in phospholipid liposomes is associated with interactions among the hydrophobic head groups, but the precise structure of the bilayer is not well defined [51]. Cholesterol has a vital function in the lipid compartment by providing rigidity to the lipid bilayer, reducing the permeability of hydrophilic substances across the liposomal membrane, and stabilizing the liposome. In the presence of cholesterol, phosphatidylcholine enhances liposome permeability and may facilitate liposome–cell fusion [52].

Numerous studies have explored the impact of optimizing the phospholipid–cholesterol ratio to enhance the efficiency of drug encapsulation and ensure its stable release. Researchers have demonstrated how adjusting this ratio affects the stability of liposomes. For example, Pereira et al. (2016) conducted a study examining the influence of lipid composition and purification on the encapsulation efficiency of docetaxel-loaded liposomes. This research specifically investigated how different lipid compositions influenced the loading and physicochemical properties of these liposomes. The liposomes were produced using the lipid-film hydration technique, succeeded by extrusion chromatography to remove unencapsulated substances. Various phospholipid and cholesterol compositions were used, along with different drug-to-lipid ratios. The research found that an increase in lipid content led to an approximately 95% encapsulation efficiency. Conversely, when the lipid content was low and the drug content was high, the encapsulation efficiency decreased to about 40% [53]. Joseph et al. (2018) created liposomes with Lornoxicam for local delivery using the thin-film hydration technique. The optimization involved varying the phospholipid and cholesterol content as independent variables. The dependent variables of the study included drug entrapment efficiency and in vitro release. A polynomial equation was used to analyze the impact of the independent variables on these outcomes. The results indicated that the optimal formulation, containing 45% cholesterol and 80% phospholipids, achieved a maximum entrapment efficiency of 98%. The optimized liposomes had a particle size of 156 nm and provided a sustained release over 8 h. Thus, adjusting the phospholipid–cholesterol ratio is crucial in liposome optimization studies [54]. Saraswat and Maher optimized quercetin-loaded liposomes by varying lipid compositions to assess the in vitro cytotoxic effects of quercetin [55]. Using the thin-film hydration technique followed by sonication, they obtained three lipid combinations. The liposomes with 3% PEG had a phosphatidylcholine–cholesterol ratio of 67:30, those with 5% PEG had a ratio of 65:30, and those with 7% PEG had a ratio of 63:30. The study demonstrated that the highest encapsulation efficiency was 90%, representing the liposomes containing 3% PEG and a phosphatidylcholine–cholesterol ratio of 67:30. This indicates that variations in the phospholipid–cholesterol ratio significantly improve the drug loading and release properties. Therefore, optimizing the lipid ratio is very important for enhancing these characteristics [55].

## 3. Liposome Preparation Methods

When choosing the method for preparing liposomes, various factors are taken into account, such as the physicochemical properties of the liposome constituents and the substance to be loaded, the concentration and toxicity of the substance to be loaded, the environment in which the liposomes will be dispersed, the desired half-life, applicability, and the costs for large-scale clinical purposes [50,56].

The main objectives in developing a liposome formulation method are to obtain monodisperse particles exhibiting a limited size distribution, to achieve the desired degree of lamellarity, to ensure the efficient encapsulation of the drug, and to maintain long-term colloidal stability. Conventional methods typically involve dissolving lipids in a volatile organic solvent and then mixing this solution containing an aqueous phase. The use of an organic solvent can alter the chemical characteristics of the active substances, potentially affecting the stability or toxicology of the nanostructured formulation. Thus, several steps are followed for the preparation of liposomes:

- Dissolving the lipids in an organic solvent.
- Evaporating the organic solvent to obtain a lipid film.
- Hydrating the lipid film with an aqueous medium, accompanied by agitation or mixing.
- Minimizing the dimensions of the liposomes and/or modifying their lamellarity.
- Post-formulation processing, including purification and sterilization.
- Characterizing the final nanoformulation product.

**Figure 1:**
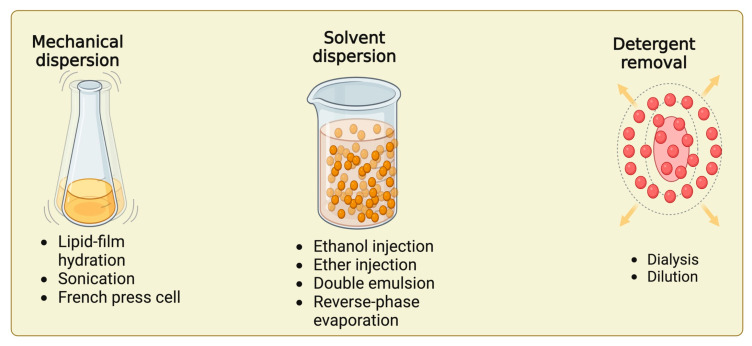
Liposomes preparation methods [62] (created with BioRender.com).

Liposomes can be prepared using conventional or advanced methods; thus, depending on the chosen method, the dimensions, composition, morphology, lamellarity, and stability of the liposomes will be influenced (Figure 1) [4,57]. Conventional methods include mechanical dispersion, solvent dispersion, and detergent removal. However, newer methods for preparation have also emerged, namely liposome extrusion and the freeze–thaw method of liposomes.

In addition to the traditional thin-film hydration method, a variety of other techniques have been developed and utilized for the preparation of second- and third-generation liposomes, including solvent injection, reverse-phase evaporation, and microfluidic methods [42].

The second generation of liposomes is typically prepared using well-established methods, such as thin-film hydration, ethanol injection, and reverse-phase evaporation, with further modifications and optimization to achieve the desired characteristics [58].

One of the key methods for the preparation of third-generation liposomes is the use of microfluidic technology. Microfluidic systems allow for the precise control and manipulation of the physical and chemical conditions during the self-assembly process of liposomes, enabling the fine-tuning of their structural parameters, such as size, lamellarity, surface properties, and even the incorporation of stimuli-responsive lipids or other functional components [59].

The third generation of liposomes has more complex internal architectures and enhanced physical stability, enabling the delivery of a wide range of therapeutics, including the recent breakthrough in mRNA-based vaccines. Owing to their versatility and adaptability, these liposome-inspired platforms have emerged as valuable tools in the treatment of a diverse array of medical conditions, with a particular focus on cancer therapy, where their ability to target and deliver drugs to tumor sites selectively has been extensively explored [60,61].

### 3.1. Conventional Methods

#### 3.1.1. Thin-Film Hydration Technique

**Figure 2:**
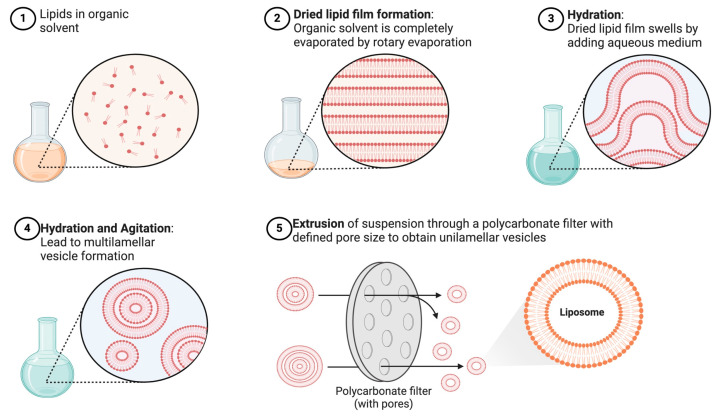
Thin-film hydration method (created with BioRender.com).

Thin-film hydration, also known as the Bangham method, is an extensively used technique for preparing liposomes. This technique involves dissolving the lipid components, usually phospholipids, in an organic solvent to create a thin lipid film by evaporating under reduced pressure [63]. This film is then hydrated with an aqueous solution, followed by vigorous agitation to facilitate the encapsulation of the drug substance, triggering the formation of liposomes [64]. This process initially produces multilamellar vesicles (MLVs) with a broad size distribution. To refine the size and reduce polydispersity, extrusion or sonication steps are employed (Figure 2).

First described in 1967, this method has served as a foundation for the synthesis of liposomes at the laboratory scale. Several factors influence liposomal characteristics, including the volume and rate of hydration. A larger volume of hydration solution tends to produce a higher quantity of multilamellar vesicles (MLVs) with a more diverse size distribution. Energetic rehydration after freeze-drying leads to the formation of MLVs, while gentle rehydration results in giant unilamellar vesicles (GUVs) [65,66].

This method is versatile, producing various vesicle structures, such as small unilamellar vesicles (SUVs), MLVs, and GUVs [67]. However, it has limitations, including a broad size distribution, a requirement for high temperatures, the potential degradation of liposomes during sonication, and a lower encapsulation efficiency [68].

#### 3.1.2. Sonication Method

The sonication method is a simple and widely used technique for producing liposomes by applying high-energy ultrasonic waves to a multilamellar vesicle (MLV) liposome solution in an inert atmosphere [66]. Key factors of this technology that determine vesicle size are given by the intensity of pressure waves and the duration of sonication [69]. There are two types of sonication techniques: bath sonication and probe sonication, both producing liposomes with similar qualities [70,71].

Bath sonication is typically used for larger volumes, and the liposome dispersion is positioned in a sterilized container with controlled temperatures or under an inert atmosphere [72]. The primary limitations of this technique include a suboptimal encapsulation efficiency, the potential degradation of phospholipids or encapsulated compounds, and a high polydispersity index [73]. Although sonication is commonly used to form small unilamellar vesicles (SUVs) with diameters ranging from 15 to 25 nm, it may not be optimal when precise physical properties of liposomes are required [67,74,75]. However, bath sonication offers superior control over operational parameters [76].

For high-energy applications involving small volumes, such as those with a high lipid content or a viscous aqueous phase, a probe sonicator is typically used [77]. This method involves directly immersing the tip of the sonicator into the liposome dispersion, which can generate significant local heating due to energy dissipation at the tip. To mitigate this, the vessel is placed in an ice bath during sonication. However, this process can lead to the hydrolysis of over 5% of the lipids within an hour. Additionally, the use of a probe sonicator can result in the loss of titanium particles from the probe tip, thereby contaminating the solution. The contamination issue can be managed by purification through ultra-centrifugation to form small unilamellar vesicles (SUVs) [78,79,80]. It is crucial to assess the impact of probe sonication on the drug substance entrapment efficiency, especially since this technique is often used for homogenizing liposomal formulations [81].

#### 3.1.3. French Press Cell Method

This technique is based on a high-pressure mechanism to create homogeneous unilamellar liposomes of intermediate sizes, ranging from 30 to 80 nm, and can handle volumes between 1 and 40 mL [82]. In terms of stability, the liposomes produced by this method are superior to those obtained through sonication. However, a significant drawback is the increased cost of the pressure cell required for the process. Despite this, the method produces liposomes of better quality compared to those produced by sonication [83].

#### 3.1.4. Solvent Injection Method

This method involves injecting phospholipids that have been previously dissolved in an organic solvent. This mixture is then injected into an aqueous solution including the active substance at a temperature higher than the boiling point of the solvent, allowing the solvent to evaporate and form liposomal vesicles [73,84,85]. Ethanol and ether are the main solvents used for the preparation of liposomal nanoformulations [86].

In the ethanol injection method, phospholipids solubilized in ethanol are quickly introduced into preheated distilled water or a TRIS-HCl buffer. When ethanol is diluted under a critical concentration in the aqueous solution, it promotes the self-assembly of lipids. The rapid dilution of ethanol facilitates the precipitation of lipid molecules and the subsequent formation of planar bilayer segments that encapsulate the aqueous phase [84]. As the ethanol evaporates, the lipid fragments fuse, leading to the creation of closed unilamellar vesicles. The volume of ethanol used is essential for the outcome; if it does not exceed 7.5% of the total volume of the formulation, small unilamellar vesicles (SUVs) are produced. However, if ethanol is injected rapidly into an excess buffer solution, multilamellar vesicles (MLVs) result. Residual ethanol is removed using a dialysis membrane, and concentration adjustments can be made through nitrogen gas pressure filtration [64]. This method allows for the formation of both large unilamellar vesicles (LUVs) and SUVs. Finally, ethanol is eliminated using a rotary evaporator at decreased pressure at a temperature of 40 °C. It is crucial to comprehend that in this mechanism, the formation of liposomes is driven not by the injection system itself but by the dispersion of a small amount of an organic phase miscible with water into a larger aqueous phase. This dispersion triggers the self-assembly of phospholipids into liposomes [87]. The size of the resulting liposomes is influenced by the lipid concentration and injection speed. The disadvantages of this method, including the low encapsulation efficiency for hydrophilic compounds, relatively low lipid solubility in ethanol, and the limited lipid concentration in the final solution, are due to the high ethanol content [88].

In the ether injection technique, lipids dissolved in ether or a mixture of diethyl ether/methanol are gradually introduced into an aqueous phase, including the components intended for encapsulation. This aqueous phase is heated to a temperature of 55–65 °C to evaporate the solvent from the liposomal formulation [66]. Further elimination of the organic solvent at decreased pressure promotes the formation of large unilamellar vesicles (LUVs). Injecting a lipid ether solution into the aqueous phase leads to the formation of small unilamellar vesicles (SUVs) [89]. A key advantage of this technique over the ethanol injection method is the more efficient removal of the organic solvent, resulting in concentrated liposomal solutions with a high encapsulation efficiency. However, the method has limitations, including the high polydispersity of the resulting liposomes (ranging from 60 to 200 nm) and the exposure of active or therapeutic compounds subjected to organic solvents and high temperatures, which may affect the safety and stability of the liposomal formulation [90].

#### 3.1.5. Double Emulsion Technique

This technique involves several key steps. First, phospholipids are dissolved in an organic solvent to create a lipid solution. This solution is then mixed with an aqueous solution to form a water-in-oil (W/O) emulsion, in which water droplets are dispersed in the lipid organic solvent phase. Next, this W/O emulsion is introduced into a second aqueous solution, leading to the formation of a double emulsion (W/O/W), where the initial water droplets are encapsulated in larger water droplets. The organic solvent is ultimately eliminated by evaporation, resulting in the formation of an aqueous liposomal suspension [91].

This technique is known for its high encapsulation efficiency, making it particularly effective for entrapping hydrophilic compounds in liposomes, compared to liposomes produced by the injection method [92]. It is most commonly used to obtain large-sized liposomes [93,94].

#### 3.1.6. Reverse Phase Evaporation Technique

This technique shares similarities with the lipid-film hydration method, but it has some differences. Phospholipids and cholesterol are solubilized in a solvent. After evaporation using a rotary evaporator, a thin film is formed at the bottom of a round-bottom flask. Unlike the lipid-film hydration technique, in this method, the thin film is redissolved in an organic solvent, usually diethyl ether or diisopropyl ether [95].

An aqueous solution including the drug substance is added to the redissolved lipid phase, followed by sonication to produce a two-phase system in which liposomes are formed. The organic solvent is then eliminated under reduced pressure, transforming the system into a viscous gel. The residual solvent can be eliminated through dialysis, centrifugation, or passage through a Sepharose column [96,97,98]. To reduce the size, multiple extrusions can be performed, with the desired size distribution achieved by adjusting the pore dimension of the polycarbonate membrane and the number of extrusions [99].

The reverse phase evaporation technique is effective for encapsulating macromolecules, including proteins and nucleic acids [100]. However, potential disadvantages include a residual organic solvent that can influence the chemical or biological stability of the drug-encapsulated liposomes [101].

#### 3.1.7. Dialysis Method

The dialysis method for detergent removal in the preparation of liposomes involves several steps to achieve the formation of large unilamellar vesicles (LUVs) with minimal detergent contamination [102]. Lipids are solubilized together with a surfactant in an organic solvent. Thus, after evaporating the solvent, a lipid film is formed, which is then hydrated, resulting in a solution of mixed micelles containing both lipids and detergent [103]. To remove the surfactant, dialysis, adsorption chromatography, or dilution techniques are used [75].

As the surfactant is removed, the lipid molecules gradually associate and reorganize into larger vesicles, ultimately forming LUVs. This method is advantageous because it produces liposomes with a uniform size distribution and minimizes the presence of residual detergent, which can be crucial for certain applications [104].

However, the dialysis method can be time consuming and may require optimization to ensure complete detergent removal without compromising the integrity of the liposomes. Additionally, the efficiency of detergent removal and the final dimensions of the liposomes can be influenced by factors such as the initial concentration of the detergent, the type of detergent used, and the properties of the dialysis membrane [105].

#### 3.1.8. Dilution Technique

The dilution technique for detergent removal in the preparation of liposomes is a simple technique used to form vesicles by gradually reducing the detergent concentration. The following steps are typically followed:

- Lipid solubilization: Initially, the lipids are solubilized in an aqueous solution with a detergent at a concentration above the critical micelle concentration (CMC). This leads to the formation of mixed micelles composed of both lipids and detergent molecules.
- Dilution process: The solution containing the micelles is subjected to gradual dilution with a detergent-free buffer. As more buffer is added, the detergent concentration decreases, falling below its CMC.
- Destabilization of micelles and formation of liposomes: As the detergent concentration drops below the CMC, the micelles destabilize, leading to the aggregation of lipid molecules and the formation of vesicles. This aggregation results in the spontaneous formation of liposomes.
- The final formation of liposomes: The dilution process continues until the detergent is sufficiently reduced, allowing the liposomes to form completely. These liposomes typically arrange into structures such as small unilamellar vesicles (SUVs) or large unilamellar vesicles (LUVs) depending on the dilution conditions and the types of lipids used.
- Optional purification: after the dilution process, any remaining detergent molecules can be removed through additional purification steps, such as dialysis or gel filtration, to ensure that the liposomes are free from detergent residues.

The dilution technique is relatively simple and does not need specialized equipment. It also tends to produce liposomes with a uniform size distribution [106]. However, achieving complete detergent removal and optimizing conditions for liposome formation may require careful control of the dilution process [107].

### 3.2. Advanced Methods

#### 3.2.1. The Extrusion Method

The extrusion method is advantageous because it produces liposomes with consistent and controllable size distributions, which is critical in various applications, especially in the medical field. It is a relatively simple process, making it suitable for both laboratory and industrial-scale liposome production. The resulting multilamellar vesicles (MLVs) are subjected to the extrusion process to obtain uniformly sized liposomes. This involves passing the MLV suspension through polycarbonate membranes with defined pore sizes using an extruder. Extrusion is typically performed multiple times, with the number of passes depending on the desired size and uniformity of the liposomes [108,109].

Recent studies have highlighted the effectiveness of the extrusion method in producing high-quality liposomes for various applications, including drug delivery, vaccine development, and gene therapy [110]. 

#### 3.2.2. Liposome Freeze–Thaw Technique

The freeze–thaw technique is a common technique used in the preparation and processing of liposomes to achieve the encapsulation of substances and improve vesicle homogeneity. This process involves several steps:

- Preparation of lipid suspension: Lipids are dispersed in an organic solvent to form a homogeneous solution. This solution is then dried, usually by rotary evaporation, to create a thin lipid film. The film is subsequently hydrated with an aqueous buffer, resulting in the formation of multilamellar vesicles (MLVs).
- Initial freezing: The lipid suspension is rapidly frozen by immersing the container holding the suspension in a liquid nitrogen bath or a carbon dioxide–ethanol ice mixture. Rapid freezing leads to the formation of ice crystals, which can disrupt the lipid bilayers of the MLVs.
- Thawing: The frozen lipid suspension is then slowly thawed at room temperature or in a water bath. The thawing process allows the ice crystals to melt, causing the disrupted lipid bilayers to fuse and form larger unilamellar vesicles (LUVs) or to reform into MLVs with an improved encapsulation efficiency.

The freeze–thaw cycle is commonly repeated multiple times (up to 10 cycles) to ensure thorough mixing and promote the formation of uniformly sized liposomes. Each cycle involves freezing the suspension again followed by thawing. After completing the freeze–thaw cycles, the liposomal suspension can undergo further processing, including extrusion or sonication, to achieve the desired size and lamellarity [111].

The freeze–thaw method is advantageous because it is relatively simple and does not require specialized equipment. It enhances the encapsulation efficiency of hydrophilic substances. However, the method may require optimization of the number of freeze–thaw cycles and careful handling to prevent the degradation of sensitive compounds [112].

Recent studies have demonstrated the effectiveness of the freeze–thaw method in improving the encapsulation and stability of liposomes, making it a valuable technique in the field of liposomal drug delivery and other applications [113].

#### 3.2.3. The Microfluidic Technique

The microfluidic technique has emerged as a highly precise approach for liposome production, offering significant advantages over traditional methods such as thin-film hydration. By using microchannels to direct fluid flow and control mixing at a microscale, microfluidics facilitates the self-assembly of lipid molecules into liposomes upon contact with aqueous buffers. This self-assembly is driven by polarity changes that prompt lipids to form stable vesicles [114]. The technique enables the fine-tuning of critical parameters, such as the flow rate and flow rate ratios, to achieve consistent liposome sizes and a high encapsulation efficiency [115]. This precise control over liposome uniformity and stability is especially beneficial for drug delivery applications, where reliable dosing and pharmacokinetics are paramount. The method is particularly suited for encapsulating diverse therapeutic molecules, including hydrophilic and hydrophobic drugs, proteins, and nucleic acids, by integrating them into respective solvent phases. This process enables continuous, one-step liposome production with minimal variability in particle size—ranging from thirty nanometers to several hundred nanometers—eliminating the need for further size-standardization steps [116]. This advancement in microfluidic liposome preparation underscores the technique’s potential to enhance the reproducibility and scalability of liposomal drug delivery systems for pharmaceutical and clinical applications.

## 4. Advantages and Disadvantages of Liposomes

**Figure 3:**
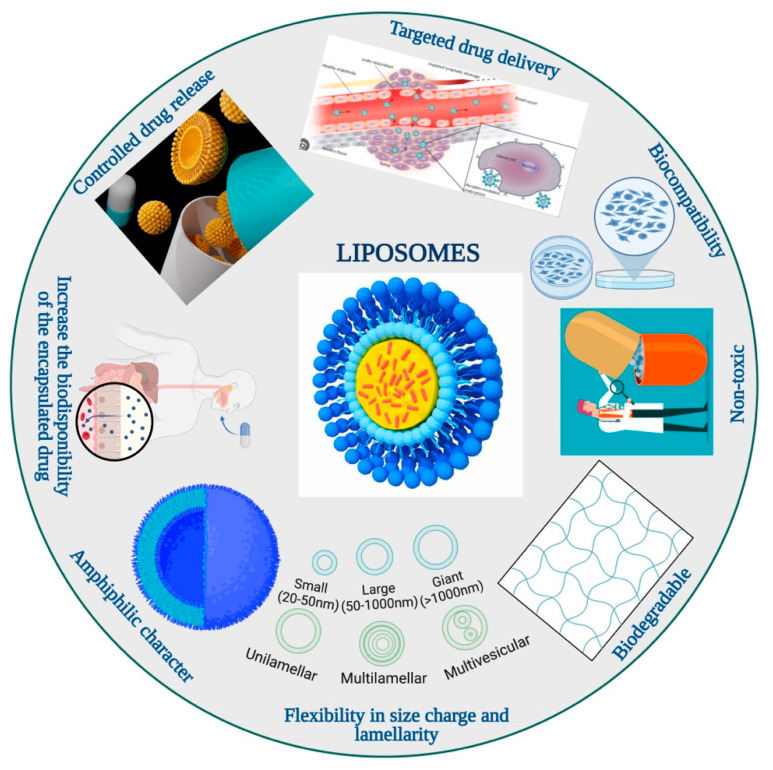
Advantages of liposomes (created with BioRender.com).

When used as drug carriers, liposomes have the following advantages: increased solubility of hydrophobic drugs, enhanced stability of drugs in vivo, targeted delivery, prolonged release time of therapeutic agents, reduced absorption of therapeutic agents by normal tissues, and decreased side effects of therapeutic agents to some extent (Figure 3) [117,118,119].

Liposomes help decrease the toxicity of encapsulated agents and can provide increased efficacy and a higher therapeutic index [120].

The key advantage of active targeting is its ability to reach lesions that are inaccessible to passively targeted nanomedicines, such as micrometastases with a weak enhanced permeability and retention (EPR) effect [121]. This capability is crucial in preventing cancer relapse by effectively targeting these small, hard-to-reach metastatic sites [122].

Extrinsic factors influencing the vasculature in drug delivery include blood circulation, organ clearance, tissue penetration, cellular uptake, and drug release. After liposomes are injected into the bloodstream, they immediately interact with blood components like proteins and nucleases, potentially altering their properties or degrading their payload, such as nucleic acids [123]. PEGylation, a widely FDA-approved strategy, enhances nanomedicine’s circulation time, improving its chances of extravasating into diseased tissue [124,125]. However, PEGylation can also reduce interactions with target tissues or cells, a challenge known as the “PEG dilemma” [126]. Additionally, PEG’s potential immunogenicity can accelerate clearance, leading to the exploration of alternatives like zwitterionic polymers and polyoxazolines [127]. Mima et al. (2017) provide an outstanding approach to reducing the immune response against PEGylated liposomes, focusing on the reduction in anti-PEG antibodies that typically emerge with repeated dosing. The study explores how the integration of gangliosides into the PEGylated liposome membrane attenuates the anti-PEG IgM response by promoting B cell tolerance. This ganglioside modification not only minimizes PEG immunogenicity but also helps preserve the liposomes’ therapeutic efficacy over multiple administrations. These findings highlight ganglioside incorporation as a promising approach to improve the biocompatibility and longevity of PEGylated liposome therapeutics, suggesting significant potential for clinical applications requiring repeated treatment [128].

Liposomes smaller than 30 nm have been shown to penetrate effectively into hypo-permeable tumors, where larger particles may struggle to reach due to the dense and poorly vascularized tissue structure. This smaller size allows for better tissue infiltration and drug delivery in challenging tumor environments [121].

Diseased tissues possess a unique pathophysiological microenvironment due to metabolic reprogramming, which results in altered redox conditions, acidity, hypoxia, and specific protein expression [129]. These characteristics present new opportunities for developing stimuli-responsive and actively targeted liposomes [130]. For example, high oxidative stress, commonly seen in tumors and brain disorders, enables the use of nanocarriers containing reactive oxygen species (ROS)-cleavable units like boronic esters and thioketal groups, allowing for selective therapeutic delivery [131,132]. Actively targeted nanomedicines involve decorating nanocarriers with affinity ligands that engage specific receptors, enhancing tissue retention and cellular uptake. Commonly used ligands include small molecules, antibodies, peptides, and aptamers, all of which can improve bioavailability [133].

**Figure 4:**
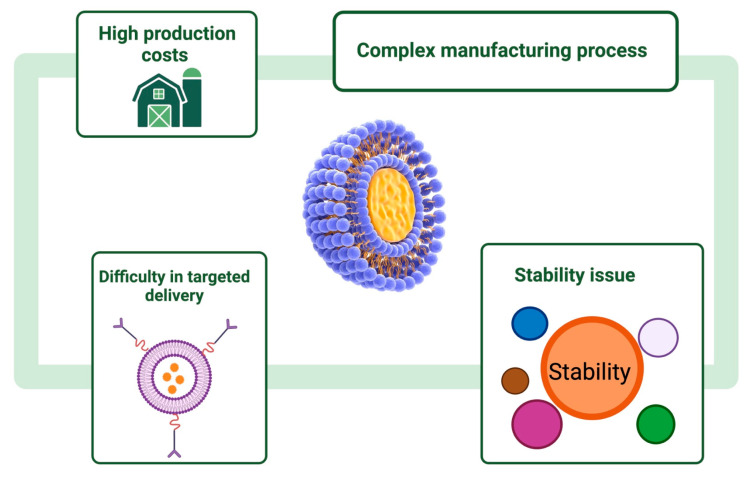
Disadvantages of liposomes (created with BioRender.com).

Although research interest in the production of liposomes has increased, several shortcomings need to be addressed: hydrolytic and oxidative degradation of phospholipids changes in the half-life of the drug and high production costs [134] (Figure 4).

The disadvantage of liposomes formed with unsaturated lipids is their susceptibility to oxidation, which can lead to the degradation of their structure [135]. In addition, both saturated and unsaturated lipids can undergo hydrolysis, resulting in the release of fatty acids, which can affect the stability and efficacy of liposomes as drug delivery systems [136,137,138]. These phenomena reduce the lifetime of liposomes and can negatively influence their therapeutic performance.

As a solution to overcome these disadvantages, it was found that if polymers are incorporated into the lipid bilayers, they can strengthen the bonds in the membrane of liposomes, and they become more resistant to chemical, enzyme, and immune reactions [139,140,141]. Polymers that are used to increase the stability of liposomes usually include hydrophobic polymers, which can integrate into the liposome structure and thus improve their physicochemical characteristics. These polymers can help stabilize lipid membranes through interactions with the aliphatic portions of phospholipids, thereby reducing the tendency for liposomes to fuse or degrade. Examples of such polymers are PEG, polyamides, polymers based on acrylic acid, and synthetic polymers [142]. Another way would be for the polymer to complex with cholesterol or other substances and thus join the lipid bilayer.

## 5. Strategies to Overcome Biological Barriers to Improving Drug Delivery of Liposomes

Liposomes can be prepared in various forms for drug delivery, including suspensions, aerosols, semisolids like gels and creams, or as dry powders, all of which can be administered through different routes.

One of the major challenges in the field of drug delivery today is improving the transport of drugs across the various biological barriers (BBs) they encounter between the site of administration and the location where they need to exert their therapeutic effects. These biological barriers hinder the accumulation of liposomes at targeted disease sites, thus reducing the effectiveness of treatments for conditions such as cancer and inflammation [143]. While these barriers play a crucial role in protecting the body by preventing harmful pathogens and substances from entering, they also complicate the delivery of drugs to the intended areas [144]. Several key barriers, including the blood–brain barrier (BBB) [121], blood–cerebrospinal fluid barrier (BCSFB), blood–lymph barrier (BlyB), blood–air barrier (BAB), stromal barrier (SB), blood–labyrinth barrier (BLaB), blood–retinal barrier (BRB), and placental barrier (PB), are located around specific tissues and organs [145]. The BBB, in particular, presents a significant challenge, especially for drugs targeting the central nervous system (CNS) in the treatment of neurodegenerative diseases and brain cancers. Other critical barriers, like the BRB, are encountered when delivering drugs to the eye [146,147,148]. These barriers act as protective systems, selectively allowing only certain essential molecules, such as nutrients and minerals, to pass through [149]. Various strategies are being explored to overcome these obstacles, with localized drug administration being a simple and effective approach for some drugs.

Upon systemic administration, regarded as the most ensuring way for these transport systems, liposomes are often identified as foreign entities by the body and are subsequently taken up by cells of the mononuclear phagocyte system (MPS), particularly the Kupffer cells located in the liver and spleen [150]. While this mechanism is beneficial for targeting drugs to these specific cells, it generally limits the ability of liposomes to be used for other purposes, such as targeted drug delivery. For site-specific targeting, ligands can be added to the liposome surface to bind to receptors overexpressed on diseased cells [151]. To overcome the issue of rapid MPS clearance, research has focused on developing liposomes that can avoid quick uptake. As a result, lipid formulations that extend liposome circulation time in the bloodstream have been discovered, with PEG-coated or sterically stabilized liposomes being among the most common.

Local delivery bypasses the need to cross major barriers and offers advantages like increased bioavailability, reduced side effects and toxicity, and, in some cases, lower costs.

**Figure 5:**
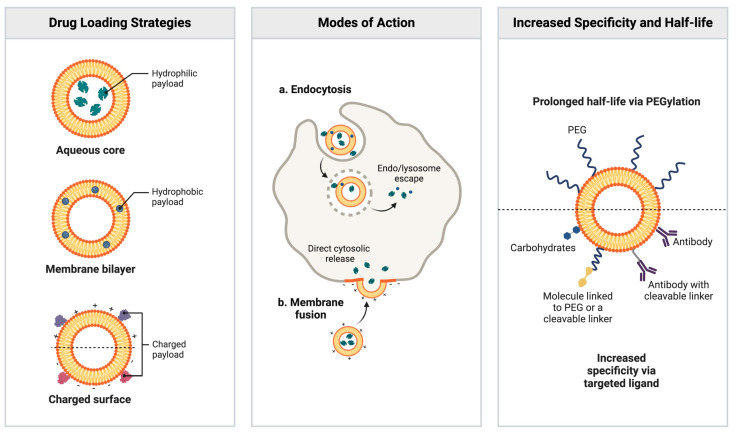
Liposomes drug delivery (created with BioRender.com).

By modifying carrier properties such as shape, surface chemistry, and responsiveness, drug carriers can more effectively penetrate tissues, bypass immune responses, and enhance cellular uptake [152]. One widely used approach to extend the circulation time of nanoparticles is PEGylation, where polyethylene glycol (PEG) is grafted onto the liposomes [153,154] (Figure 5). Long-circulating or PEGylated liposomes offer significant therapeutic benefits by altering the biodistribution and pharmacokinetics of drugs compared to their free form [125]. This creates a hydrating layer that prevents protein adsorption and clearance by the immune system, as shown in the case of PEGylated liposomal doxorubicin, which significantly extended the drug’s lifetime [155].

Doxil was the first commercially successful product based on PEGylated liposomes. It demonstrated the benefits of this technology by extending the drug circulation time and reducing side effects, particularly in cancer treatments [156]. A notable example is the significantly decreased cardiotoxicity observed with liposomal doxorubicin versus the free drug [157]. Additionally, the slow release of drugs from these liposomes, which remain intact for extended periods after administration, can lead to less frequent dosing requirements, further enhancing patient outcomes [158]. While other materials like poloxamer and polysaccharides have been tested, PEG remains the most popular. In one study, PEGylated liposome–polycation–DNA nanoparticles showed low liver uptake and high tumor accumulation, demonstrating the effectiveness of this strategy in evading immune clearance [159].

Intranasal (IN) delivery is a promising route for both locally and systemically acting drugs. The nasal mucosa’s permeability allows for high drug concentrations in the blood while avoiding first-pass metabolism and enzymatic breakdown, improving patient compliance [160]. IN delivery has garnered attention for its potential in direct nose-to-brain (N-B) drug transport, bypassing the blood–brain barrier (BBB) [161,162]. Various carriers, including polymeric liposomes, micelles, and nanoemulsions, have been explored for brain-targeted drug delivery via this route. The IN delivery of liposomal drugs for Alzheimer’s disease (AD) treatment, such as Donepezil (DNP) and Rivastigmine (RV), has shown promising results in rat models, with studies demonstrating significantly higher plasma levels of DNP compared to free drug administration, confirming the potential of liposomes for nose-to-brain drug delivery [163]. In another study, Quetiapine fumarate (QTF), an antipsychotic drug, was formulated in liposomes and demonstrated higher brain concentrations [164] and an improved brain-to-plasma ratio compared to QTF dispersions [165]. Additionally, an intranasal (IN) administration of Risperidone-loaded liposomes in Wistar albino rats showed increased brain drug levels compared to systemic administration. Among the tested formulations, PEGylated liposomes achieved the highest brain bioavailability after IN delivery, further confirming the effectiveness of direct nose-to-brain drug transport [166].

Another study with Senicapoc-loaded liposomes dispersed in a thermosensitive hydrogel (Pluronic F-127) showed enhanced drug bioavailability and prolonged residence time on the ocular surface. Hydrogels provided superior drug retention compared to conventional topical viscous solutions [167].

Liposomal (LIP) dermal delivery systems are primarily used for treating skin-related conditions such as acne, psoriasis, infections, inflammatory diseases, and skin cancer. A common approach is embedding liposomal drugs in gels to improve skin retention and bioavailability, which is a major challenge for dermal delivery. To address the limited skin permeability of traditional liposomes, ultradeformable liposomes (UDLs), such as Transfersomes^®^, were developed [168,169]. These advanced liposomes use surfactants to enhance membrane flexibility, which increases their ability to penetrate the skin’s stratum corneum without compromising their structure [170]. UDLs provide controlled, continuous transdermal drug delivery, improving patient convenience and efficacy by avoiding gastrointestinal factors and first-pass metabolism [171]. UDLs have demonstrated superior skin permeation for drugs like diclofenac, dexamethasone, and insulin compared to conventional liposomes [172,173,174,175,176]. For example, Zedoary turmeric oil (ZTO) and tretinoin (TRE)-loaded LIPs, incorporated into a Carbopol gel matrix, showed enhanced drug penetration into hair follicles and increased skin retention in mice compared to conventional gels. In vivo studies demonstrated that the LIP gel was more effective in treating psoriasis in a dose-dependent manner [177].

## 6. Liposome Characterization

The efficacy of liposomes as drug delivery vehicles is tightly connected to their supramolecular, microscopic, and nanoscopic structure. It is essential to evaluate the quality of liposomes and obtain quantitative measurements to compare different batches of liposomes.

It is known that vesicle size affects pharmacokinetics, tissue distribution, the clearance of liposomes, absorption and hepatic accumulation, tissue diffusion, and renal excretion depending on particle size.

To ensure their function in vitro and in vivo, liposomes must be fully characterized after preparation and before application to evaluate their physical and chemical properties. Size, size distribution (measured by the polydispersity index, PDI), surface charge (measured by the Zeta potential), shape, lamellarity, phase behavior, entrapment efficiency, and in vitro release are the most studied properties of liposomes [178]. Various techniques are used to assess liposome size and size distribution, such as microscopy or static or dynamic light scattering [179].

Traditional liposomes have demonstrated restricted stability in vitro.

Second-generation liposomes were engineered to extend blood circulation and improve in vivo stability, therefore addressing this problem [180]. They can be characterized by their size distribution, Zeta potential, cryo-electron microscopy or Atomic Force Microscopy (to visualize the morphology and structure of the liposomes), cryo-electron microscopy or Atomic Force Microscopy to visualize the morphology and structure of the liposomes, high-performance liquid chromatography (to quantify the encapsulation efficiency and drug loading of the liposomes), scanning electron microscopy (SEM), liposome stability, in vitro release studies (to evaluate the drug release kinetics from the liposomal formulation), quantification of PEG or targeting ligand conjugation efficiency, polydispersity index [30], and pharmacokinetic analysis using animal models (to determine the circulation time, biodistribution, and tissue-targeting capabilities of the stealth liposomes) [60,181].

Compared to the characterization approaches for first- and second-generation liposomes, the evaluation of third-generation liposomes typically involves a more comprehensive assessment, focusing on the effectiveness and efficiency of the targeting strategy. Characterization methods for third-generation liposomes include the evaluation of the targeting ligand conjugation efficiency, receptor-mediated binding, internalization, scanning electron microscopy (SEM), and pharmacokinetic and biodistribution properties of the liposomal formulation [182].

### 6.1. Size and Polydispersity Index

Liposomes are primarily characterized by their size and polydispersity index (PDI). It has been established that the size of liposomes is critical for inhalation and parenteral administration [183] as well as for determining the half-life of liposome circulation [184,185] because it influences various properties, including the stability, entrapment efficiency, drug release, mucoadhesion, and cellular uptake of liposomes [186]. While small-sized liposomes can circulate for an extended period in the body, large-sized liposomes are quickly eliminated from the bloodstream [187], which is why liposomes with sizes between 50 and 200 nm are used for drug delivery [99].

The PDI value indicates the sample size and the degree of heterogeneity within it, which can be monodisperse or polydisperse. The PDI can be dimensionless and scaled between 0 and 1. A PDI value less than or equal to 0.3 indicates an appropriate and homogeneous liposome structure [185], while a high value indicates a very broad distribution (heterogeneity) or possibly multiple liposomal structures in the sample [188]. The PDI is calculated using the particle size, refractive index of the solvent, measurement angle, and distribution variance.

Dynamic light scattering (DLS) and Nanoparticle Tracking Analysis (NTA) are used for analyzing nanoparticle size, especially for formulations like liposomes. Each method provides distinct insights: DLS measures size distribution based on light scattering, and NTA tracks individual particle movement to gauge both size and concentration, enhancing precision in particle characterization [189].

DLS is used to analyze the continuous movement of particles dispersed in solution (Brownian motion), resulting in light scattering. The DLS method is simple, straightforward, rapid, and reliable, capable of determining the size of liposomes in their original environment. Additionally, it can measure sizes from a few nanometers to micrometers. However, this method has certain disadvantages, including the inability to distinguish individual particles from conglomerates and increased sensitivity to identifying minute impurities (contaminants) [190].

NTA is an effective method for assessing liposome concentration and size distribution within a sample. NTA works by monitoring individual liposome particles via microscopy and evaluating their Brownian motion to determine size and concentration. This approach delivers precise particle count data in real time, facilitating a detailed examination of liposome samples, including batch consistency and particle concentration per unit volume. NTA provides an effective method for quantifying and verifying the quality of liposome formulations in both development and manufacturing contexts [191].

Another method for determining liposome size is performed using electron microscopy, which allows for the visualization of liposomes individually, thereby obtaining accurate information about all present liposomes and displaying their sizes [192].

### 6.2. Zeta Potential

In general, the net charge of particles is described in terms of the surface charge or Zeta potential [193]. This characteristic of liposomes is considered a critical physical feature for controlling the electrostatic interactions among suspended particles [194]. The net charge of liposomes is determined by several critical characteristics, including their lipid composition and associated ligands, which can be negative, neutral, or positive. Generally, liposomes with low or no charge tend to aggregate over time as there are no forces preventing flocculation.

### 6.3. Liposomes Morphology

Microscopy is the most accurate technique to identify the morphological characteristics of liposomes [193]. Electron microscopy techniques, such as Transmission Electron Microscopy (TEM), allow for the direct study of liposomes as individual particles.

Another option to circumvent these constraints is the use of cryo-TEM. This method preserves the initial state of liposomes and reduces the distortion or contraction of shape by rapidly freezing them with liquid nitrogen and then observing them directly in a controlled setting. However, cryo-TEM is usually more effective with samples of smaller sizes as larger particles may be removed during the preparation process.

Atomic Force Microscopy (AFM) appears to be a technique for analyzing liposomes directly in their original environment without sample processing. It is considered a rapid, efficient, and non-invasive procedure [183]. The primary benefit of this approach over electron microscopy is the high resolution of three-dimensional micrographs, reaching the order of nm-Å [195].

Lamellarity is another property that can influence liposomal applications due to its effect on the entrapment efficiency and the drug release profile.

### 6.4. Entrapment Efficiency

Entrapment efficiency is described as the ratio of the quantity of drug contained in liposomes (the encapsulated drug) to the total amount of drug used in the manufacture of the liposomes (both incorporated and unincorporated drugs). The entrapment capacity of liposomes is essential for determining the potential of the vesicles as drug delivery systems. This capacity is expressed as the entrapment efficiency (EE) and refers to the percentage of the drug incorporated into the aqueous environment relative to the initial amount of drug used for entrapment:

Entrapment Efficiency (%) = (mass of incorporated drug/mass of total drug) × 100

A thorough examination of liposomal properties can enable the development of liposomal formulations with optimal EE and control over drug release. The composition of the liposome, the method of its fabrication, and the rigidity of the lipid bilayer can all have a significant effect on the EE of a specific drug [196].

Loading capacity refers to the quantity of drug encapsulated per unit weight of the liposomes, representing the percentage of the liposome’s mass attributed to the drug. It is calculated by dividing the total amount of entrapped drugs by the total weight of the lipids expressed as a percentage (LC%) [27]:

LC% = (mass of incorporated drug/mass of lipids) × 100

Common approaches to determining the amount of drug contained in liposomes largely depend on their composition and include ultraviolet–visible (UV–VIS) spectrophotometry and fluorescence spectroscopy [183]. In addition, more complex equipment such as HPLC (high-performance liquid chromatography), UPLC (ultra-performance liquid chromatography), GC (gas chromatography), and MS (mass spectrometry) can be used to determine the amount of drug [197].

### 6.5. Drug Loading

Determining drug loading in liposomes is essential for understanding a formulation’s therapeutic potential. Drug loading indicates the amount of active drug that a liposome can carry, impacting the dosing, efficacy, and release profile of the drug [198].

Drug loading in liposomes depends on factors like the drug’s properties, lipid composition, liposome size and type, drug-to-lipid ratio, and the preparation method. Each variable affects how much drug can be encapsulated and retained within the liposome, influencing the release profile and bioavailability [199]. For instance, lipids with a stronger affinity for a drug enhance loading, while optimal liposome size and type help maintain stability [200]. Adjusting these factors allows for controlled drug release, consistent efficacy, and optimized dosing suited to the therapeutic goal. Higher loading maximizes the therapeutic effect per dose, reduces the need for frequent administration, and can minimize side effects by ensuring a controlled release. Additionally, consistent drug loading across batches is crucial for product reliability and regulatory compliance in pharmaceutical development [201].

### 6.6. In Vitro Drug Release

To evaluate the in vitro drug release profile, methods like the Franz diffusion cell, dialysis method, ultrafiltration, and centrifugation are used.

The membrane in the diffusion cell must be chosen according to the specifications of the analyzed drugs. It should be permeable and free of adsorption. At predetermined time intervals, a portion of the samples is obtained and assessed using drug quantification procedures, replacing it with a fresh receptor medium to maintain a constant volume throughout the determination period [178]. By graphing the cumulative release percentage against selected time intervals, the data are used to construct the drug release profile [202].

Dialysis method: Liposome samples are placed in a dialysis membrane bag within a buffer solution. This allows drug molecules to diffuse out while retaining liposomes inside, giving a controlled release profile [203].

Ultrafiltration and centrifugation: these methods involve physically separating free drugs from encapsulated ones by applying either a centrifugal force or a filtration barrier, facilitating the precise analysis of released drug content [204].

## 7. Clinical Applications of Liposomes

### 7.1. The Use of Liposomes with Active Substances

Liposomes have become sophisticated tools for the targeted delivery of various active substances [205] and are considered one of the most effective delivery systems discovered [206]. These lipid vesicles possess the distinctive ability to encapsulate a wide range of therapeutic agents, enabling precise delivery to specific cells or tissues [158,207,208]. The introduction of novel imaging modalities, advanced interpretation techniques, and innovative formulation methodologies for targeted delivery systems has shaped the quest to address the challenges associated with conventional liposomal formulations [209]. In studies on post-ischemic reperfusion, CDP-choline-loaded liposomes significantly improved survival rates and reduced the maturation phenomenon, which is the progressive neurodegenerative damage that can follow an ischemic stroke. Compared to free CDP-choline, the liposomal formulation provided stronger neuroprotection and significantly mitigated oxidative damage, likely due to enhanced delivery and retention within brain tissues. The liposomal carrier’s ability to protect against lipoperoxidative stress highlights its potential for improving post-stroke outcomes by effectively targeting and sustaining therapeutic action at the injury site [210]. Fresta and Puglisi (1997) showed in their study with liposomal encapsulated CDP-choline a noticeable improvement in the survival rate with regard to the free drug, ranging from 45% to 100% as a function of the duration of the ischemic event [211]. d’Avanzo et al. (2024) demonstrated the improved brain-targeting properties of functionalized liposomes in vivo, enhancing the survival rates in ischemic and reperfusion rat models, which is one of the main challenges in precision medicine to date [212].

Currently, liposomes are used in the medical field, being regarded as the most suitable carriers for the introduction of various therapeutic agents, such as anticancer drugs, anti-inflammatory drugs, antibiotics, hormones, antifungals, enzymes, and proteins, in vivo [213,214]. In general, liposomes used in medical applications range from 50 to 450 nm [215] (Table 1).

The technology for producing nanoparticles provides exceptional prospects for the pharmaceutical industry, enabling the controlled incorporation and release of diverse compounds and attaining optimal bioavailability and stability, particularly for delicate drugs. The healthcare industry offers several applications owing to nanoencapsulation and the advantages conferred by liposomes: enhanced effectiveness, superior biocompatibility, minimal immunogenicity, safeguarding of pharmaceutical agents, extended medication half-life, and reduced toxicity [236].

Cancer is a disease that contributes to mortality rates in numerous countries. Unfortunately, the effectiveness of conventional therapy for various types of malignancies is suboptimal [237]. By encapsulating chemotherapeutic agents in liposomes, their selectivity for neoplastic cells and tumor tissues can be improved via passive or ligand-mediated active targeting [238]. This leads to a reduction in the adverse effects of the drug and an increase in anticancer efficacy due to the enhanced accumulation of liposomes within tumors [239,240,241,242,243,244]. Promising enhanced accuracy and effectiveness in therapeutic interventions, this approach represents a significant advancement toward improving the prospects of patients facing this challenging disease [245,246,247]. Several FDA-approved liposomal anticancer drugs illustrate their efficacy. The first liposomal delivery system for anticancer drugs, containing doxorubicin, that received FDA approval was Doxil^®^ (also known as Caelyx^®^), introduced by Sequus Pharmaceuticals in 1995. This formulation consists of pegylated liposomal doxorubicin (DOX) developed primarily for the treatment of Kaposi’s sarcoma. Since the approval of Doxil^®^, several other liposomal systems containing cytotoxic agents have been approved for clinical use. Another pegylated liposomal DOX formulation approved by the FDA, LipoDox^®^, was produced by Sun Pharma in 2012. DaunoXome^®^, which contains daunorubicin, was the second liposomal anthracycline anticancer drug targeting the treatment of acute myeloid leukemia (AML). Myocet^®^, a non-pegylated liposomal DOX, has demonstrated a shorter half-life and reduced cardiac side effects.

ThermoDox^®^, a thermosensitive liposomal formulation of doxorubicin (DOX), is the first heat-activated drug carrier that has been used in human clinical trials [248]. At temperatures between 40 and 45 °C, the thermosensitive liposomes in ThermoDox^®^ quickly change structure to release doxorubicin (DOX) at the designed site [249]. In clinical research, ThermoDox^®^ has been utilized with radiofrequency ablation for the treatment of hepatocellular cancer. Although the OPTIMA trial failed to achieve its objectives, this is not proof that ThermoDox^®^ is unviable. Preliminary clinical data have shown that ThermoDox^®^ is feasible, safe, and effective [248]. A new phase I trial at the University of Oxford is investigating the efficacy and security of combining ThermoDox^®^ with targeted ultrasound for the treatment of unresectable pancreatic cancer. Additionally, de Maar JS et al. [250] conducted the first phase I feasibility study of ThermoDox^®^ combined with cyclophosphamide and high-intensity focused ultrasound-induced hyperthermia guided by magnetic resonance imaging in patients with stage IV breast cancer.

For the treatment of leukemia, Marqibo^®^ is used, a product containing liposomal vincristine sulfate that leads to greater accumulation in target tissues where vincristine is gradually released [227]. Onivyde^®^ (irinotecan hydrochloride) is prescribed for pancreatic adenocarcinoma and exhibits a prolonged antitumor effect [251,252]. It is presented in injectable form, containing liposomal irinotecan (IRI), and received FDA approval in 2015 for second-line therapy of pancreatic ductal adenocarcinoma. This formulation is a liposomal topoisomerase inhibitor that prevents DNA replication in cancer cells, enhancing the accumulation of IRI at the tumor site through the improved permeability and retention effect (EPR). In human colon cancer (HT29) and breast cancer (BT474 [74]) xenograft models, liposomal IRI demonstrated increased drug loading and an extended half-life compared to free IRI, resulting in significantly greater cytotoxic activity [253]. Onivyde™, combined with leucovorin and fluorouracil, is designed for patients with metastatic pancreatic adenocarcinoma who have not responded to gemcitabine-based therapy [254].

Shen et al. [255] developed a PEGylated liposomal formulation containing mannose with levamisole hydrochloride to suppress tumor proliferation. This formulation inhibits glycolysis and mitochondrial energy consumption in both cancer cells and macrophages. Moreover, when utilized alongside radiation, these liposomes not only augment the therapeutic efficacy on localized tumors but also activate the immune response to impede metastatic lesions.

In colon cancer, liposomes have been used to deliver therapeutic agents directly to neoplastic cells in the colon, enhancing efficacy and reducing the adverse effects of existing chemotherapeutics. However, many of these formulations are still in the experimental phase. Khuntawee et al. (2021) formulated liposomes with cordycepin, a potential treatment for colon cancer, to address its limitations, such as insufficient stability and limited solubility in water. In vitro studies demonstrated that liposomal cordycepin significantly suppressed the proliferation of colorectal cancer cell line HT-29, inducing apoptosis at a rate twice that of unencapsulated cordycepin [256]. Xiong et al. (2017) developed a mannosylated liposomal formulation loaded with paclitaxel for targeted delivery to mannose receptors, which are abundantly expressed in the CT26 colon cancer cell lines. The study results indicated the improved cellular uptake of mannosylated liposomes, with no observed toxicity or side effects [257].

Oral administration is the preferred method for drug delivery, especially as it facilitates easier adherence to treatment protocols for colorectal cancer (CRC) patients [258]. The main difficulty in the oral administration of liposomal preparations arises from the harsh conditions in the gastrointestinal (GI) tract. Factors such as pH level, transit time, enzymes, and microbiota can influence the stability and targeting effectiveness of these nanoparticles [259,260]. Still, the same conditions present significant opportunities for the development of liposomes designed for oral administration [261].

Intravenous (IV), intradermal (ID), intramuscular (IM), and subcutaneous (SC) administrations are among the most effective for increasing the systemic bioavailability of drugs. However, while these routes improve systemic availability, they do not necessarily ensure high concentrations of active substances, and systemic side effects may occur in CRC treatment.

Alongside established clinical liposomal formulations, a multitude of novel liposomal drug delivery technologies for oncological treatment are presently in development. Platinum nanoparticles (nano-Pt) exhibit high cytotoxicity and kill cancer cells by releasing Pt ions under low pH conditions [262]. Additionally, nano-Pt acts as a catalase-like nanozyme, making it useful as an oxygen-complementing nanomaterial to address hypoxia limitations in photodynamic therapy. Liu et al. [263] created biomimetic liposomal platinum by encapsulating nano-Pt and the photosensitizer vitepofen in macrophage membrane-coated liposomes. This liposomal delivery system achieved deeper penetration into tumor tissues and improved chemotherapy efficacy through catalyzed oxygen delivery with nano-Pt.

Vaccination is a highly affordable preventative measure against a wide range of diseases, including those caused by viral, bacterial, fungal, or parasitic infections, as well as cancerous conditions and autoimmune diseases like rheumatoid arthritis [264]. The potential of liposomes to stimulate immune responses when used as vaccine adjuvants or when associated with antigens was first demonstrated by Gregoriadis and Allison (see [265]). Since then, various types of liposomes along with virosomes have emerged as significant platforms in vaccine development. Interest in liposome-based vaccines has grown considerably, leading to the clinical approval of vaccines like Epaxal, Inflexal, and Mosquirix [266]. Liposomes offer an excellent method for delivering subunit vaccines, which consist of pathogen fragments capable of triggering an immune response. The cationic liposome formulation, 1,2-dioleoyl-3-trimethylammonium-propane (DOTAP), has been thoroughly investigated for its potential as a vaccine delivery method. Recent research has investigated the integration of liposomes with immune-stimulating ligands to enhance the creation of innovative adjuvant systems.

A primary benefit of particulate vaccines is their ability to shield antigens from enzymatic degradation by encapsulation while simultaneously providing molecular adjuvants to antigen-presenting cells (APCs), thus enhancing both cellular and humoral immune responses [267].

A vaccine has been developed to prevent and mitigate the transmission of human immunodeficiency virus (HIV) (ACTHIVE-001). This vaccine includes a native-like HIV-1 membrane combined with MPLA liposomes and is presently undergoing a phase I clinical study (NCT03961438) to assess its safety and efficacy in healthy individuals [268].

The anti-comedogenic efficacy assessment of 1% clindamycin liposomes and 1% standard clindamycin solution showed that 33.3% of patients receiving liposome treatment effectively eliminated blackheads (open comedones) without adverse effects, whereas only 8.33% of patients treated with the standard clindamycin solution had a similar outcome. Liposomal clindamycin proved effective for closed comedones, pustules, and papules [269].

### 7.2. Liposomes with Plant Extracts

Plant extracts have numerous health benefits because of their antioxidant, antimicrobial, anti-inflammatory, hepatoprotective, and neuroprotective activities [270,271]. However, they also have some disadvantages concerning high hygroscopicity, low solubility, and reduced bioavailability. To address these limitations, researchers have suggested using efficient entrapment and delivery systems, such as liposomes, which can effectively transport both hydrophilic and lipophilic phyto-constituents [272,273].

Sogut et al. observed that the solubility of fat-soluble vitamins increased when used in liposomal form [274]. Plant extracts can degrade and lose effectiveness when exposed to environmental factors. Liposomal systems provide stability against chemical and environmental fluctuations, thus enhancing the stability of these extracts. As a result, liposome-based delivery systems are used to protect components sensitive to light, pH changes, oxygen, temperature, and humidity. Various studies have demonstrated this improved stability in plant extracts using curcumin [275,276,277], vitamins, and polyphenols.

Tan et al. conducted a comparison between carotenoid-encapsulated liposomes and carotenoid solutions and found that their antioxidant activity was enhanced when carotenoids were encapsulated in liposomes. Furthermore, they validated the improvement in efficacy by combining it with minerals [278].

Curcumin is among the most frequently explored substances owing to its potent anti-inflammatory effects, but its limited solubility in water and reduced bioavailability limit its biological activity. To enhance these aspects, researchers have employed liposomal delivery systems [279,280,281]. Studies by Tiyaboonchai et al. (2007) [282], Noack et al. (2012) [283], Nayak et al. (2010) [284], Ambarsari et al. (2014) [285], and Shelat et al. (2015) [286] have shown that incorporation into liposomal systems leads to better anti-inflammatory effects, provides sustained release, and improves antioxidant capacity compared to conventional forms of these substances.

Resveratrol has anti-inflammatory effects but is restricted by poor solubility, which can be improved through encapsulation in liposomes. Jøraholmen et al. [287] found that encapsulation enhanced the anti-inflammatory activity of resveratrol. Additionally, resveratrol and epicatechin demonstrated efficacy in treating vaginal infections and inflammation due to their potent antioxidant and anti-inflammatory effects [288].

Gortzi et al. [289] investigated the antimicrobial properties of oregano extract encapsulated in liposomes. The results indicated that the liposomal formulation exhibited greater antimicrobial activity compared to the unencapsulated extract.

Cui et al. [290] explored the use of sage oil as an antibacterial agent against *Staphylococcus aureus* when encapsulated in liposomes. Their study involved the use of α-toxin, secreted by *S. aureus*, to stimulate the release of sage oil from liposomes, enhancing its antibacterial effect against *S. aureus*.

Green tea extract is rich in catechins, which have antioxidant, anti-inflammatory, and anticancer effects. Dag and Oztop (2017) observed that the use of a liposomal administration of green tea extract led to an enhancement in the oral bioavailability and therapeutic efficacy of catechins [291].

Plant extracts with reduced solubility and increased permeability are often preferred to be formulated as liposomal delivery systems (Table 2) [292].

Green tea is considered a beneficial natural drink for health due to its content of catechins, which have been shown to possess antioxidant, anticancer, and cardiovascular disease-prevention effects. Tsai and Chen [311] investigated the impact of a catechin nanoemulsion on prostate cancer using PC-3 cell cultures in vitro. Their study demonstrated that this catechin delivery system may impede the proliferation of PC-3 tumor cells by causing apoptosis. This was achieved by reducing B-cell lymphoma 2 expression and increasing cytochrome c expression, which activated caspase-3, caspase-8, and caspase-9.

Hepatic cellular carcinoma is a predominant cause of cancer-related deaths worldwide. Curcumin shows significant potential in enhancing the chemosensitivity of hepatic cellular carcinoma to chemotherapeutics by modulating various signaling pathways. To address the issue of cisplatin resistance and its limited clinical efficacy, Cheng et al. [312] developed liposomes encapsulated with cisplatin and curcumin for precise administration and release in the treatment of hepatic cellular carcinoma. In their in vitro study, the loaded liposomes exhibited better antitumor activity against HepG2 cells, largely due to high levels of intracellular reactive oxygen species under treatment. In some countries, plant extracts are recognized for their hepatoprotective properties. Among them, resveratrol and silymarin are noted for their strong hepatoprotective effects due to their ability to support liver regeneration [313].

Kildaci et al. developed and conducted an in vitro and in silico study on nanoemulsions encapsulated with linseed oil (LSO) via ultrasonic emulsification for the treatment of atopic dermatitis. The in vitro evaluation of LSO-NE demonstrated nontoxicity to the Salmonella/Ames test strain. The data indicated that these LSO-NEs exhibited increased cellular and cutaneous permeability. The topical administration of these formulations may serve as a viable therapeutic for the treatment of atopic dermatitis [314].

One study demonstrated significant variations in moisture elevation among human participants exposed to phosphatidylcholine concentrations of 0%, 10%, 28%, and 80%. The formulation containing 80% phosphatidylcholine exhibited a superior level of moisture compared to the others. The variations in phosphatidylcholine concentration across various formulations demonstrate that the moisturizing effect is attributable to phosphatidylcholine. The hydrogenated soybean phosphatidylcholine was seen to maintain the epidermal barrier and permeate into the stratum corneum, hence reducing transepidermal water loss [315].

A SOPHY liposomal gel, containing encapsulated active compounds including L-arginine, Curcuma extract, hyaluronic acid, lactic acid, and tocopherol, demonstrated a reduction in transepidermal water loss (TEWL) and an enhancement in skin hydration when assessed on healthy human volunteers over 28 days. Skin moisture levels increased by 6.3% throughout the initial seven days of the research. Skin moisture levels increased by 14.1%, 30.3%, and 33.5% during the second, third, and fourth weeks of gel treatment, respectively, with a minor erythemal index. It also provides increased resistance to bacterial infections by altering skin pH to a more acidic level [316].

## 8. Future Perspectives

Looking ahead, the future of liposome technology holds great promise, with several key trends and directions for continued development and innovation.

One emerging trend is the increasing focus on theranostic liposomes, which combine diagnostic and therapeutic capabilities within a single liposomal platform. These liposomes can be loaded with both imaging agents and therapeutic payloads, allowing for the non-invasive monitoring of drug delivery and treatment response.

Another area of active research is the development of stimuli-responsive liposomes, which are designed to release their cargo under specific environmental conditions, such as changes in pH, redox potential, or temperature [317].

Additionally, there is growing interest in multifunctional liposomes, which can incorporate multiple features, such as targeting ligands, stealth coatings, and triggered release mechanisms, to improve their therapeutic efficacy and targeting capabilities [318].

Further advancements in the manufacturing and scale-up of liposomal formulations are also expected, as the field strives to transition more liposomal-based therapies from the laboratory to the clinic [253].

Overall, the future of liposome technology holds great promise, with the potential to revolutionize the delivery of a wide range of therapeutic and diagnostic agents in medicine and beyond.

## 9. Conclusions

The primary benefits of liposomes involve control over pharmacokinetic and pharmacodynamic characteristics, enhanced bioavailability, and reduced toxic effects, allowing them to transcend the challenges of conventional therapies. Many liposomes have successfully transitioned for practical application, whilst others are undergoing different phases of clinical testing. Although many products have shown promise in preclinical studies, only those demonstrating efficacy in clinical trials will be approved for clinical use.

While plant extracts offer a wide variety of health advantages due to their strong antioxidant, antimicrobial, anti-inflammatory, hepatoprotective, and neuroprotective properties, their clinical utility is frequently obstructed by certain constraints, such as high hygroscopicity, low solubility, and reduced bioavailability. The adoption of advanced encapsulation technologies, particularly liposomal delivery systems, has become known as a viable approach to address these challenges. Liposomes, due to their capacity to encapsulate both hydrophilic and lipophilic substances, not only enhance the solubility and stability of plant extracts but also protect these bioactive components from environmental degradation, thus preserving their therapeutic efficacy. This encapsulation approach represents a significant advancement in formulating plant-based therapies, improving their bioavailability, and ensuring more controlled and sustained release, which is crucial for maximizing their clinical potential.

Understanding the precise mechanisms by which liposomes target tumor sites and release their active ingredient payloads is crucial for addressing existing challenges in cancer therapy. By employing various surface modification techniques, multifunctional nanocarriers can be created by attaching different varieties of ligands to a single carrier. In cancer treatment, these multifunctional liposomes, which provide sustained release, precise distribution, and synergistic effects via various functionalization and surface modification strategies, will be crucial.

## Figures and Tables

**Table 1:** Liposomal formulation and clinical applications.

Name	Active Ingredient	Advantages	Indication	References
Onyvide^®^	Irinotecan, fluorouracil, folinic acid	According to clinical and statistical studies, patients treated with this liposomal formulation increased the median global survival rate to 6.1 from 4.2 months and progression-free survival to 3.1 from 1.5 months	Pancreatic adenocarcinoma	[216]
LEP-ETU	Paclitaxel	The average liposome size was about 150 nm before and after lyophilization and the EE% was greater than 90%, and stability studies showed that the liposomes after lyophilization were much more physically stable for at least 12 months at 2–8 °C and chemical for at least 12 months at 25 °C	Ovarian cancer	[215,217]
Inflexal V^®^	Inactivated hemagglutinin of A or B influenza virus	The effectiveness of the vaccine in preventing the risk of hospitalization for influenza or pneumonia in elderly patients was more than 95%	Influenza	[218]
Visudyne^®^	Verteporfin	High and rapid accumulation in atherosclerotic plaque, short half-life, few side effects	Ocular histoplasmosis	[219]
Depocyt	Cytarabine	Slow (for 14 days) and continuous release of BC at the level of CRF	Neoplastic meningitis	[220]
DaunoXome^®^	Daunorubicin	The results of the phase III randomized clinical trial showed that the CR was 64% and the probability of survival was 38%. Reduced cardiotoxicity	Leukemia	[184,221,222]
Lipusu^®^	Paclitaxel	After testing the efficacy of LP compared to NLP, it was observed that the overall response rate in the case of LP was 47% compared to 46% in the case of NLP, the disease control rate was 73% compared to 71%, and AR of nausea, vomiting, hypersensitivity were less with LP	Gastric, ovarian, and lung cancer	[223,224]
Doxyl/Caelyx	Doxorubicin	Slow release and reduced systemic toxicity	Breast and ovarian cancer	[225]
Myocet^®^	Doxorubicin with cyclophosphamide	Increased therapeutic index of L-DOX, reduced cardiotoxicity, and better tolerance than free DOX	Metastatic breast cancer	[226]
Marquibo^®^	Vincristine	In mouse xenograft studies, L-VCR was much better tolerated and showed improved antitumor activity and higher drug delivery capacity in the target tissue compared to NL-VCR	Acute lymphoblastic leukemia	[227]
Lipo-dox^®^	Doxorubicin	Lower ARs (myelosuppression, cardiotoxicity, and alopecia) compared to free DOX	Ovarian and breast cancer	[228,229]
CPX-351 (Vyxeos^®^)	Daunorubicin and cytarabine	The overall survival rate of patients treated with the liposomal formulation increased to 9.6 months compared to 5.9 months for patients treated with a non-liposomal formulation	Acute myeloid leukemia	[230,231]
AmBisome^®^	Amphotericin B	Low renal toxicity, the survival rate of mice infected with *Aspergillus fumigatus* given 10 mg/kg LAB was 60%	Meningitis, invasive fungal infections	[232]
Fungisome	Amphotericin B	It has antifungal activity demonstrated in vitro, by the microdilution method, on 262 fungal isolates	Aspergillosis, systemic candida	[233]
DepoDur^®^	Morphine sulfate	Provides prolonged release of BC, much better postoperative pain control compared to NL-M	Postsurgical pain	[234]
Exparel^®^	Bupivacaine	Provides prolonged release of BC, prolonging the duration of sciatic nerve block in rats up to 240 min	Postsurgical pain, nerve block	[235]

Legend 1: EE%—entrapment efficiency, BC—bioactive compound, CRF—cerebrospinal fluid, LP—liposomal paclitaxel, NLP—non-liposomal paclitaxel, AR—adverse reactions, L-DOX—liposomal doxorubicin, L-VCR—liposomal vincristine, NL-VCR—non-liposomal vincristine, LAB—liposomal amphotericin, NL-M—non-liposomal morphine.

**Table 2:** Liposomes with plant extracts.

Compound	Particle Size (nm)	Biological Activity	Application	References
Quercetin	200	Antioxidant.	Hepatoprotection against arsenic-induced oxidative stress.	[293,294]
Resveratrol	Unreported	Anti-inflammatory antioxidant, antimutagenic, antitumor.	Improves neuroprotective efficacy.	[295,296,297,298]
Propolis flavonoids	Unreported	Antimicrobial, anti-inflammatory, antioxidative hepatoprotective, and immunostimulatory properties.	Enhances effectiveness.	[299]
*Salvia officinalis*	˂80	Antimicrobial, astringent, cognitive-enhancer, antidepressant, and hypoglycemic.	Improves antioxidant efficacy.	[300]
Silymarin	145–329	Hepatoprotective.	Improves absorption of silymarin when taken orally.	[301,302,303]
*Armoracia rusticana* ethanolic extract	72.01 ± 0.57	Antimicrobial antioxidant, anti-inflammatory chemopreventive, gastroprotective, and hypocholesterolemic properties.	Enhanced stability of the extract formulation.	[304]
Arbutin	179–212	Antioxidant, anti-inflammatory properties.	Improves skin-brightening properties.	[305]
Carotenoids	95–140	Antioxidant, anti-inflammatory effect, photoprotection, eye health.	Increased activity and solubility.	[278]
Coenzyme Q10	68	Antioxidant, cardiovascular, anti-aging, neuroprotective effect, immune system support.	Improves solubility, stability.	[306]
Retinol	98	Antioxidant, anti-aging, reproductive effect.	Improves solubility, stability.	[307]
Curcumin	Not reported	Antioxidant, anticancer, antimicrobial, anti-inflammatory, neuroprotective, antidiabetic effect.	Better controlled release.	[308]
Hibiscus extract	<46	Antioxidant, antimicrobial, anti-inflammatory, antidiabetic, diuretic, cardiovascular effect.	Reduced fat oxidation within the liposomal systems.	[309]
Neem extract	141.6	Antifungal in seborrheic dermatitis.	Synergic effect of ketoconazole with neem extract.	[310]
